# PHY34 inhibits autophagy through V-ATPase V0A2 subunit inhibition and CAS/CSE1L nuclear cargo trafficking in high grade serous ovarian cancer

**DOI:** 10.1038/s41419-021-04495-w

**Published:** 2022-01-10

**Authors:** Amrita Salvi, Alexandria N. Young, Andrew C. Huntsman, Melissa R. Pergande, Melissa A. Korkmaz, Rathnayake A. Rathnayake, Brittney K. Mize, A. Douglas Kinghorn, Xiaoli Zhang, Kiira Ratia, Markus Schirle, Jason R. Thomas, Scott M. Brittain, Claude Shelton, Leslie N. Aldrich, Stephanie M. Cologna, James R. Fuchs, Joanna E. Burdette

**Affiliations:** 1 Department of Pharmaceutical Sciences, College of Pharmacy, University of Illinois at Chicago, Chicago, IL 60607 USA; 2 Division of Medicinal Chemistry and Pharmacognosy, College of Pharmacy, The Ohio State University, Columbus, OH 43210 USA; 3 Department of Chemistry, University of Illinois at Chicago, Chicago, IL 60607 USA; 4 Department of Biomedical Informatics, The Ohio State University, Columbus, OH 43210 USA; 5 Novartis Institutes for BioMedical Research, 181 Massachusetts Avenue, Cambridge, MA 02139 USA

**Keywords:** Drug development, Target identification

## Abstract

PHY34 is a synthetic small molecule, inspired by a compound naturally occurring in tropical plants of the *Phyllanthus* genus. PHY34 was developed to have potent in vitro and in vivo anticancer activity against high grade serous ovarian cancer (HGSOC) cells. Mechanistically, PHY34 induced apoptosis in ovarian cancer cells by late-stage autophagy inhibition. Furthermore, PHY34 significantly reduced tumor burden in a xenograft model of ovarian cancer. In order to identify its molecular target/s, we undertook an unbiased approach utilizing mass spectrometry-based chemoproteomics. Protein targets from the nucleocytoplasmic transport pathway were identified from the pulldown assay with the cellular apoptosis susceptibility (CAS) protein, also known as CSE1L, representing a likely candidate protein. A tumor microarray confirmed data from mRNA expression data in public databases that CAS expression was elevated in HGSOC and correlated with worse clinical outcomes. Overexpression of CAS reduced PHY34 induced apoptosis in ovarian cancer cells based on PARP cleavage and Annexin V staining. Compounds with a diphyllin structure similar to PHY34 have been shown to inhibit the ATP6V0A2 subunit of V(vacuolar)-ATPase. Therefore, ATP6V0A2 wild-type and ATP6V0A2 V823 mutant cell lines were tested with PHY34, and it was able to induce cell death in the wild-type at 246 pM while the mutant cells were resistant up to 55.46 nM. Overall, our data demonstrate that PHY34 is a promising small molecule for cancer therapy that targets the ATP6V0A2 subunit to induce autophagy inhibition while interacting with CAS and altering nuclear localization of proteins.

## Introduction

High grade serous ovarian cancer (HGSOC) is the most lethal form of ovarian cancer with the lowest 5-year survival rate [1]. Despite progress from new therapies, most patients develop chemoresistance, necessitating the investigation of novel drug leads [2–4]. V-ATPase inhibitors have been a target for cancer therapy for decades, however, a compound needs to be selective to specific subunits to target the tumor without systemic effects [5]. Of the V-ATPase subunits, the V0A2 subunit contributes to cisplatin resistance in ovarian cancer, acidification required for MMP-regulated metastasis, and modulation of immune cell populations [6–8]. Compounds with a diphyllin core have been shown to inhibit v-ATPase function [9–12]. HTP-013, a member of the lignan natural product family was shown to bind directly to the ATP6V0A2 subunit leading to cell toxicity and inhibition of lysosomal acidification [12]. Late-stage autophagy inhibitors are effective in clinical trials, but the only approved drug in this class is hydroxychloroquine which blocks autophagosome-lysosome fusion, but does not directly abolish lysosomal acidification [13].

Natural product drug discovery has played a role in the production of >70% of all small molecule anti-cancer agents developed to date, including paclitaxel, one of the first-line treatments for HGSOC [14]. Phyllanthusmins (PHYs), a class of diphyllin anti-cancer compounds originally isolated from the *Phyllanthus* genus, inspired the generation of the synthetic analog PHY34 [15–17]. PHY34 was cytotoxic against ovarian cancer cell lines in vitro and reduced HGSOC tumor burden in vivo through late-stage autophagy inhibition and apoptosis [17]. Ovarian cancer in particular has been shown to have the most disrupted autophagy pathway, as well as compensatory proteolytic pathways [18]. The purpose of this study was to elucidate PHY34’s molecular target in HGSOC cells, which we found may involve inhibition of nucleocytoplasmic transport via the cellular apoptosis susceptibility (CAS) protein, as well as inhibition of ATP6V0A2 subunit.

CAS (also known as CSE1L or XPO2) has many roles, including its action as a nuclear exporter of α-importins [19], as well as functions in proliferation, apoptosis, and cell division [20], epigenetic silencing [21], and microvesicle formation [22]. α-importins are transported from the nucleus into the cytoplasm by CAS for their use in nuclear import [23]. CAS is essential for cancer proliferation and survival, as shown in a genome-scale CRIPSR-Cas9 essentiality screen of 342 cancer cell lines [24]. The CAS gene is located in a known cancer amplification hot spot on chromosome 20q13, and studies on ovarian cancer patients have shown it may be amplified in ~70% of HGSOC patients [25]. CAS is highly expressed in various cancer types, including ovarian [26, 26], colorectal [27], testicular [28], breast [29], hepatocellular [30, 31], lung [32], bladder [33], oligodendroglial [34], thyroid [35], esophageal [36], and lymphomas and melanomas [37]. Its expression has been shown to correlate with poor clinical outcomes, such as chromosomal instability [38]. CAS knockdown increases cell death and/or decreases proliferation [27, 28, 39, 40], decreases chemoresistance [39], and causes cell cycle arrest [28, 39, 40]. The cytotoxic effect of CAS knockdown is reported to be specific to cancer cells, sparing non-tumorigenic cells in vitro [39].

In this study, we identified PHY34 as an autophagy inhibitor that functions by blocking ATP6V0A2 subunit, as well as interacting with CAS to affect nuclear-cytoplasmic transport. Modulation of autophagy was also mediated by inhibition of ATP6V0A2, a subunit of the membrane-associated domain of V-ATPase by PHY34. To date, no small molecules have been reported to have in vivo efficacy as V0A2 inhibitors or as CAS inhibitors.

## Results

### PHY34 interacts with members of the nucleocytoplasmic transport pathway

**Fig. 1:**
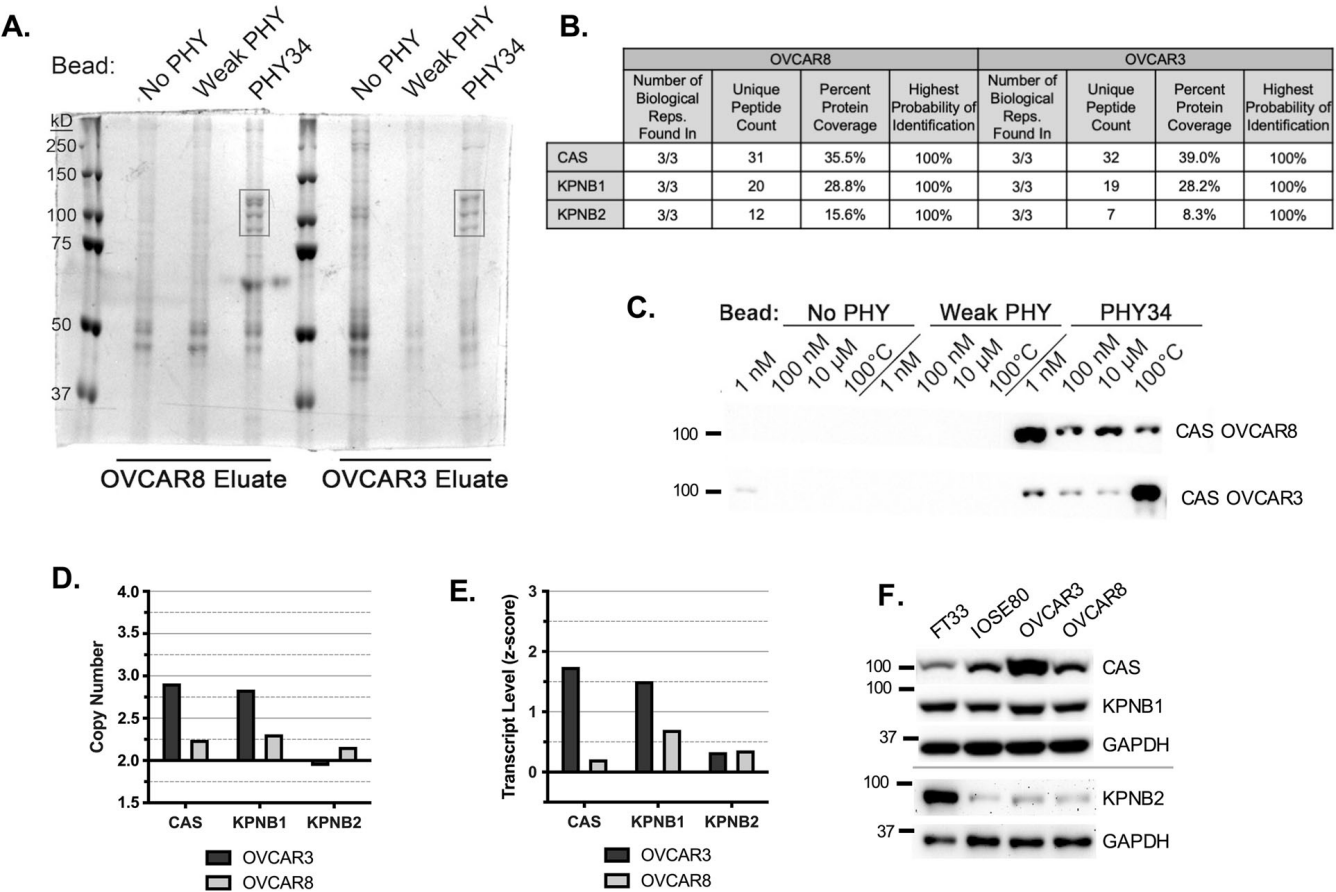
PHY34 interacts with members of the nucleocytoplasmic transport pathway. **A** Representative image of SDS-PAGE separating pulldown eluates from two HGSOC cell lines, OVCAR8 and OVCAR3, stained with Coomassie blue to reveal potential target proteins appearing in PHY34 beaded samples (black box) but not negative controls (only beads), NO PHY and Weak PHY, which were subjected to mass spectrometry for identification. **B** Selected hits and corresponding mass spectrometry data. **C** Immunoblot of CAS using eluates from competition pulldown. **D** Relative DNA copy number of targets: CAS, KPNB1, and KPNB2 in HGSOC cell lines obtained from CellMiner. **E** Relative mRNA expression of targets: CAS, KPNB1, and KPNB2 in HGSOC cell lines obtained from CellMiner. **F** Representative immunoblots assessing relative protein amounts of targets: CAS, KPNB1, and KPNB2 in HGSOC cell lines and two non-tumorigenic cell lines, FT33 and IOSE80.

Our previous studies outlined PHY34 as a late-stage autophagy inhibitor; however, the molecular target of PHY34 was unclear [17]. In order to identify cellular targets of PHY34, it was immobilized on photocrosslinker beads, along with PHY65, which served as a negative control based on its micromolar toxicity (“weak PHY”, Supplemental Fig. 1A-B). Beads were incubated with lysates from OVCAR8 and OVCAR3. A set of bands on the SDS-PAGE gel located near 100 kD appeared only in PHY34 bead eluates in 3 biological replicates in both cell lines (Fig. 1A). Bands were identified by mass spectrometry and analyzed with Gene Ontology (GO) pathway analysis, which identified the nucleocytoplasmic pathway (Fig. 1B, Supplemental Fig. 1C). Figure 1B displays the protein coverage (Supplemental Fig. 1D), which was the highest for CAS, followed by KPNB1 and KPNB2.

The interaction between PHY34 and CAS was confirmed with competition pulldown, where the assay was modified by adding increasing concentrations of free PHY34. CAS was confirmed by immunoblotting (Fig. 1C), and concentrations of free PHY34 as low as 1 nM in elution buffer competed bound protein off the PHY34 bead. The flow-through showed a corresponding decrease in targets in the PHY34 samples (Supplemental Fig. 1E).

The CellMiner database was used to evaluate DNA copy number and gene expression of PHY34 target proteins in HGSOC cell lines (Fig. 1D, E) [41]. OVCAR3, which requires higher doses of PHY34 than OVCAR8 to induce cell death, had higher levels of CAS than OVCAR8 at the DNA, mRNA, and protein level (Fig. 1F). HGSOC cell lines had more CAS than non-tumorigenic FT33 from the fallopian tube epithelium (FTE) and an immortalized ovarian surface epithelial cell line (IOSE80).

### CAS is a promising drug target as evidenced by patient sample analysis

**Fig. 2:**
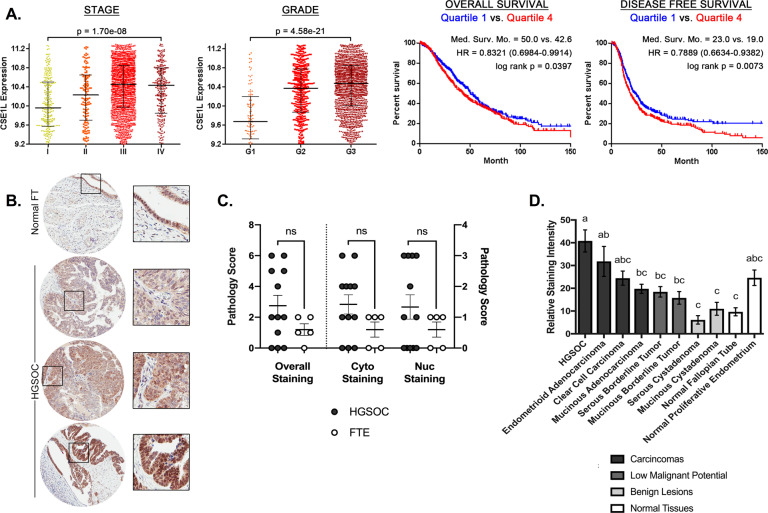
CAS is a promising drug target as evidenced by patient sample analysis. **A** Cancer Science Institute of Singapore Ovarian Cancer Database (CSIOVDB) ovarian cancer patient data of CAS expression correlated to stage and grade (*n* = 3431), overall survival (*n* = 1868), and disease-free survival (*n* = 1516). The 25% of patients with lowest expression (Quartile 1, blue) is compared to the 25% of patients with highest expression (Quartile 4, red) for survival data. HR = hazard ratio. **B** Representative images for TMA staining for CAS. **C** TMA scoring by two independent pathologists, averaged. Statistical comparisons between HGSOC and FTE pairs were assessed with unpaired *t*-tests. ns = not significant. **D** Quantification of staining intensity for TMA samples (*n* = 6–12 patients per group). All groups were compared to each other with a one-way ANOVA with Tukey’s multiple comparison. Means that do not share a letter are statistically different (*p* < 0.05).

The Cancer Science Institute of Singapore Ovarian Cancer Database was used to assess CAS expression and HGSOC progression and survival. CAS expression correlated with increasing stage and grade of disease, and patients in the highest quartile of CAS expression had worse overall and disease-free survival than those in the lowest quartile (Fig. 2A). Furthermore, CAS expression was higher in high grade serous tumors compared to normal FTE (Fig. 2B, C). We analyzed HGSOC patient data for CAS, KPNB1, and KPNB2 expression in the Cancer Genome Atlas (TCGA) based on copy number alteration (CNAs, *n* = 579), gene expression (RNA Seq V2 RSEM z-scores, *n* = 308), and protein levels (Clinical Proteomic Tumor Analysis Consortium z-scores, *n* = 174) [42, 43]. CAS was the most highly amplified target by CNA (Supplemental Fig. 2A). Oncomine datasets comparing HGSOC to normal tissue controls also supported CAS as the most differentially expressed target (Supplemental Fig. 2B) [44]. To confirm that CAS was increased in HGSOC, we measured CAS levels using immunohistochemistry on tissue microarray (TMA) samples purchased from The Cancer Human Tissue Network (CHTN Ovarian Cancer Survey OvCa2), which included patient samples from various ovarian cancer subtypes (Fig. 2B, Supplemental Fig. 2C). Samples were scored by pathologists (Fig. 2C) and quantified using ImageJ software (NIH) (Fig. 2D) and HGSOC had the highest levels of CAS. CAS expression correlated with chromosomal instability in breast cancer [38]. Therefore, the CIN70 score was calculated [45] using TCGA Affymetrix U133 microarray HGSOC samples (*n* = 531) and highly correlated with CAS expression (Supplemental Fig. 2D). KPNB1 and KPNB2 expression did not correlate with clinical endpoints, such as stage, grade, or survival of ovarian cancer or chromosomal instability. Hence, investigation of the role of CAS in HGSOC was prioritized.

### PHY34 has a distinct mechanism of action from known nucleocytoplasmic transport inhibitors

**Fig. 3:**
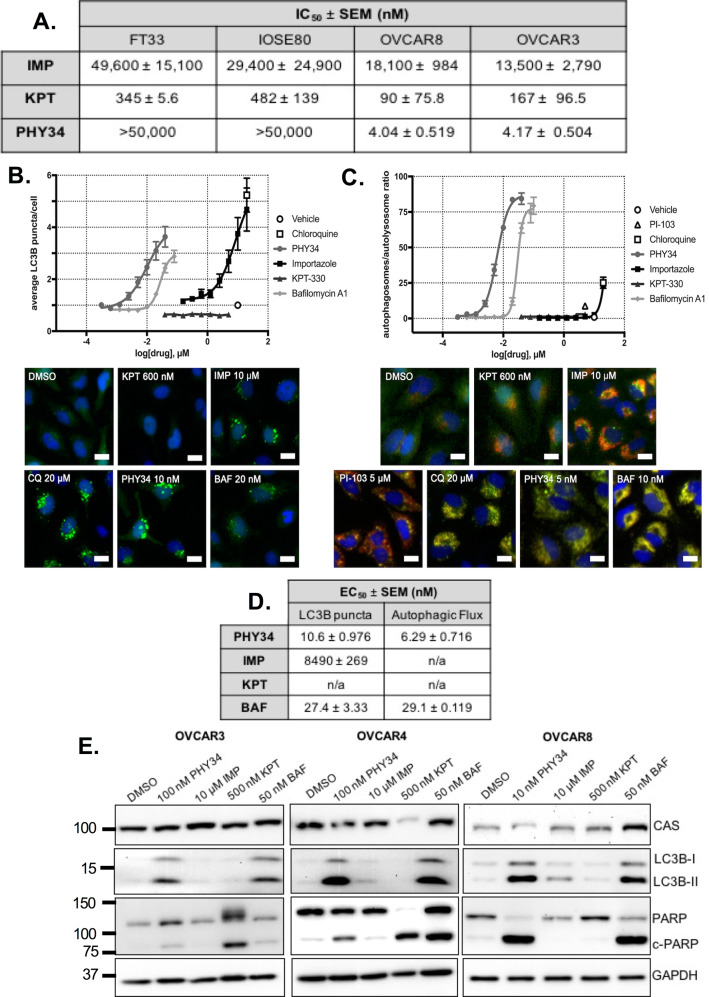
PHY34 has a distinct mechanism of action than known nucleocytoplasmic transport inhibitors. **A** IC_50_ values generated from dose-response curves measuring cell viability after 72 h treatment in HGSOC cell lines, OVCAR8 and OVCAR3, and non-tumorigenic cell lines, FT33 and IOSE80. SEM = standard error of the means. **B** Dose-response curves and representative images (scale bar = 20 μm) for LC3B puncta assay conducted in eGFP-HeLa cells after 4 h treatment. **C** Dose-response curves and representative images (scale bar = 20 μm) for autophagic flux assay conducted in mCherry-eGFP-HeLa cells after 24 h treatment. **D** EC_50_ values generated from LC3B puncta and autophagic flux assays. SEM = standard error of the means. **E** Representative immunoblots for levels of CAS, autophagy biomarker (LC3B-II), and apoptosis biomarker (c-PARP) after PHY34 treatment in HGSOC cell lines, OVCAR3, OVCAR4, and OVCAR8.

To determine if PHY34 had a unique mode of action, it was compared to inhibitors of the nucleocytoplasmic transport pathway: importazole (KPNB1 inhibitor) and KPT-330 (XPO1 inhibitor, selinexor) [46]. Dose-response curves showed that importazole had micromolar potency, while KPT-330 had mid-nanomolar potency (Supplemental Fig. 3, Fig. 3A). However, PHY34 treatment was specific for HGSOC cell lines, with low nanomolar potency against OVCAR8 and OVCAR3 and undetectable IC_50_ up to the highest dose tested at 50 μM in non-tumorigenic, FT33 and IOSE80.

Since we previously confirmed PHY34 as a late-stage autophagy inhibitor [17], importazole and KPT-330 were tested for their ability to modulate autophagy through LC3B puncta and autophagic flux assays. Importazole was able to increase puncta at a higher concentration than PHY34 or bafilomycin A1 (Fig. 3B–D). Both inducers and late-stage autophagy inhibitors may increase the number of LC3B puncta in this assay. In an autophagic flux assay, only late-stage autophagy inhibitors will cause an accumulation of autophagosomes (yellow due to co-localization of fluorophores from mCherry-eGFP-LC3B), while autophagy inducers will promote the formation of autolysosomes (red) in which the GFP signal is quenched in the acidic environment. Importazole displayed increased flux through the pathway, designating it as an autophagy inducer (Fig. 3C, D). KPT-330 did not modulate autophagy. As shown in Fig. 3C, D, PHY34 was significantly more potent than bafilomycin A1 at inhibiting autophagy (PHY34, ED_50_ = 6.29 ± 0.716 nM vs bafilomycin A1, ED_50_ = 29.1 ± 0.119 nM).

Results were confirmed in HGSOC cell lines by immunoblotting for LC3B-II protein, as well as for cleaved PARP (c-PARP) (Fig. 3E). PHY34 and bafilomycin A1 displayed the highest c-PARP and LC3B-II levels. Levels of CAS were unchanged after inhibitor treatment (Fig. 3E).

### CAS overexpression reduces PHY34 induced cell death and CAS knockdown alters response to PHY34 in HGSOC cells

**Fig. 4:**
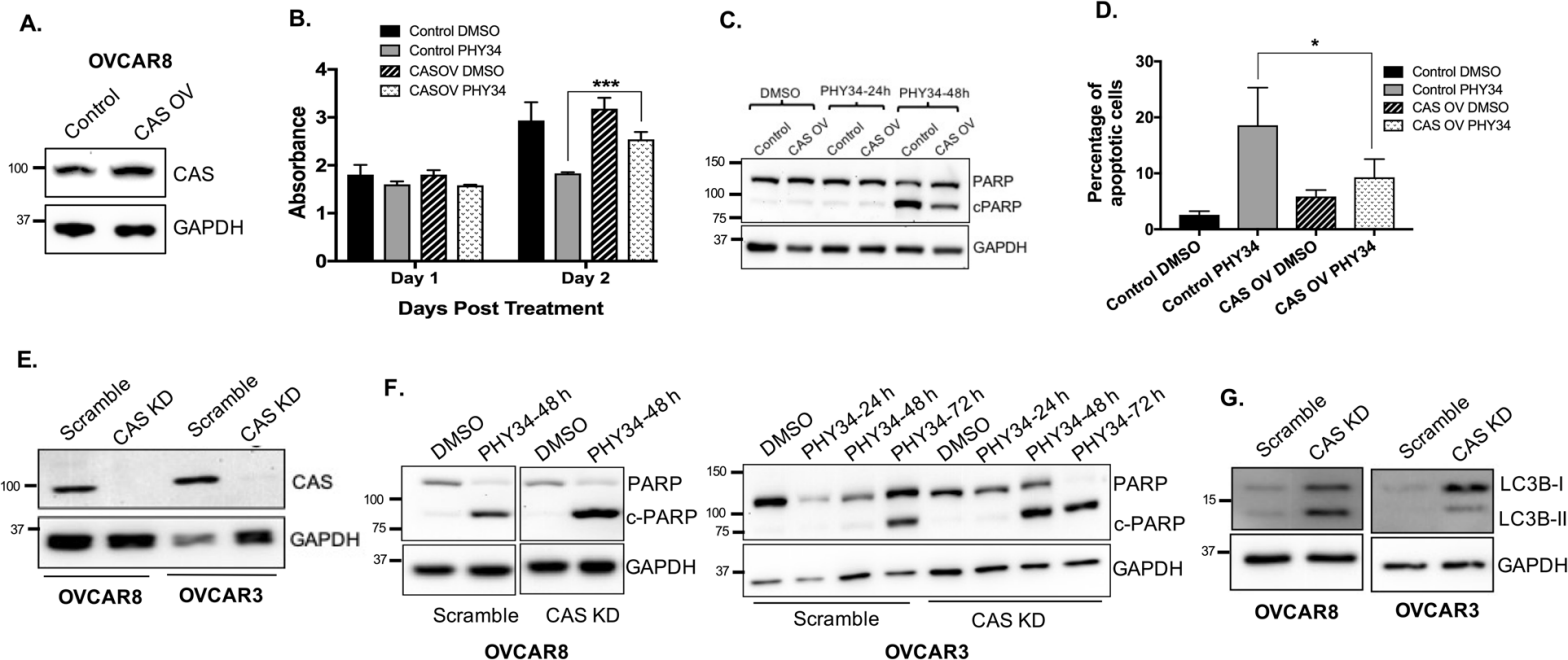
CAS overexpression reduces PHY34 induced cell death and CAS knockdown modulates autophagy in HGSOC cells. **A** Representative immunoblot for levels of CAS in CAS overexpressing OVCAR8 cells (CAS OV) generated by lentiviral transduction. Cells expressing empty plasmid were used as control. **B** OVCAR8 cells with CAS overexpression were treated with vehicle (DMSO) and PHY34 (10 nM) for 48 h. Cell viability was measured by SRB assay. Each experiment was performed in three biological replicates, and data represent mean ± SEM. Statistics were generated with Student’s *t*-test. ***p < 0.001. **C** Representative immunoblot for PARP expression in CAS overexpressing OVCAR8 cells (CAS OV) treated with vehicle (DMSO) and PHY34 (10 nM). **D** Cells were treated with vehicle (DMSO) and PHY34 (10 nM) for 48 h, stained with Annexin V-FITC (AV) and propidium iodide (PI), and analyzed by Nexcelom Cellometer. Percentages of early (AV^+^, PI^−^) apoptotic cells were quantified. Each experiment was performed in three biological replicates, and data represent mean + /-SEM. Statistics were generated with one-way ANOVA with Dunnett multiple comparisons with vehicle control within each group. *p < 0.05. **E** Representative immunoblot for CAS expression in OVCAR3 and OVCAR8 cells expressing scramble shRNA (Scramble) and CAS shRNA (CAS KD). **F** Representative immunoblot for PARP expression in OVCAR8 and OVCAR3 cells with stable knockdown of CAS (CAS KD) and treated with PHY34 (10 nM and 100 nM in OVCAR8 and OVCAR3 cells, respectively). **G** Representative immunoblot for LC3B expression in OVCAR3 and OVCAR8 cells expressing scramble shRNA (Scramble) and CAS shRNA (CAS KD).

To test the role of CAS in PHY34 mediated cytotoxicity, CAS was overexpressed in OVCAR8 cells by lentiviral transduction (Fig. 4A). Cell viability assays were performed and showed that CAS overexpression reduced PHY34 mediated cytotoxicity (Fig. 4B). We confirmed this by immunoblotting for c-PARP and found that CAS overexpressing cells show a reduction in c-PARP expression as compared to control cells treated with PHY34 (Fig. 4C). Annexin V/Propidium iodide staining demonstrated that CAS overexpression significantly reduced the percentage of apoptotic cells compared to control cells after PHY34 treatment (Fig. 4D). These data suggest that when CAS is overexpressed, PHY34 is less effective at inducing apoptosis.

To determine if loss of CAS would modulate sensitivity to PHY34, we performed lentiviral shRNA mediated stable knockdown of CAS (Fig. 4E). Reduction of CAS increased sensitivity of cells towards PHY34 by inducing apoptosis at an earlier timepoint in OVCAR3 cells and with more PARP cleavage in OVCAR8 cells compared to control cells (Fig. 4F). Since CAS has no reported association with autophagy, we investigated if its loss had an effect on LC3B expression. Knockdown of CAS increased LC3B-II expression using immunoblot and (Fig. 4G), uncovering a previously unknown result of CAS loss. These data suggest that knockdown of CAS mimics aspects of PHY34 treatment in HGSOC cells in terms of apoptosis induction and increased LC3B-II expression as shown previously [17].

### PHY34 treatment and CAS knockdown leads to cargo mislocalization

**Fig. 5:**
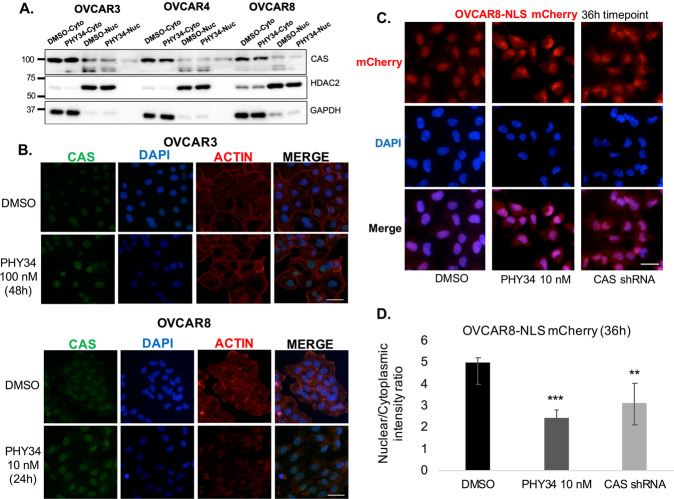
PHY34 treatment and CAS knockdown leads to cargo mislocalization. **A** Representative immunoblots for CAS expression in OVCAR3, OVCAR4, and OVCAR8 cells using nuclear and cytoplasmic fractions of PHY34 treated cells. **B** Representative immunofluorescence images for CAS localization in OVCAR3, OVCAR4, and OVCAR8 cells treated with PHY34. DAPI and actin were used as nuclear and cytoplasm stains. Scale bar = 20 μm. **C** Representative images of OVCAR8-NLS-mCherry cells treated with PHY34 (10 nM) and CAS shRNA lentivirus at 36 h timepoint. Scale bar = 20 μm. **D** Nuclear and cytoplasmic intensities were quantified using ImageJ software (NIH). Ratios of the nuclear-to-cytoplasmic intensity of PHY34 treated (10 nM) and CAS shRNA samples were analyzed with unpaired *t*-test to vehicle control. **p < 0.01, ***p < 0.001.

Next, we sought to understand how interaction of PHY34 with CAS might impact expression and function of CAS. As shown in Fig. 3E, CAS expression was unchanged in HGSOC cells treated with PHY34. Using immunofluorescence, we found that PHY34 did not change CAS localization (Fig. 5A, B). This suggests that PHY34 may inhibit CAS function by disrupting nuclear-cytoplasmic trafficking. As the major function of CAS is the recycling of α-importins to the cytoplasm for use in nuclear import of nuclear localization sequence (NLS)-tagged protein [19], we explored the localization of NLS-mCherry with fluorescence imaging. A stable NLS-mCherry OVCAR8 cell line was generated and live cells were imaged after treatment with PHY34 (Fig. 5C). Intriguingly, fluorescent signal increased in both nuclear and cytoplasmic compartments after 36 h treatment with PHY34 or CAS shRNA lentiviral particles. The net shift of NLS-mCherry was into the cytoplasmic compartment, as shown by a decrease in nuclear-to-cytoplasmic ratio of signal (Fig. 5D). This provided functional evidence that nuclear trafficking is impacted by PHY34 and CAS knockdown.

### Quantitative proteomics shows that PHY34 changes subcellular localization of multiple proteins

**Fig. 6:**
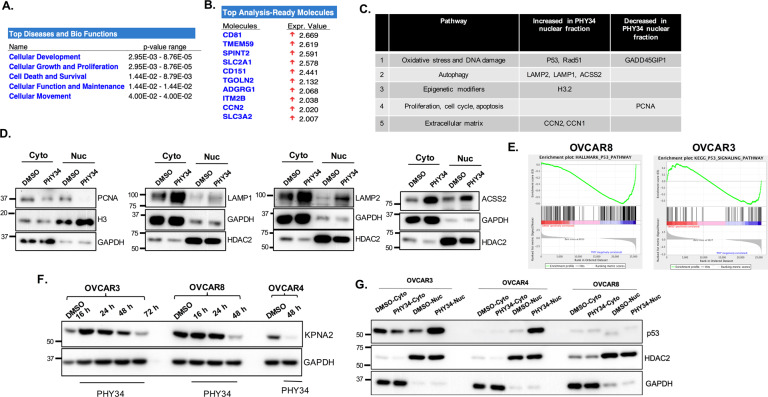
Quantitative proteomics shows that PHY34 changes subcellular localization of multiple proteins. **A** Ingenuity pathway analysis of nuclear-cytoplasmic proteomics showing top differentially regulated pathways and biological functions after PHY34 (100 nM) treatment in OVCAR3 cells. **B** Ingenuity pathway analysis of nuclear-cytoplasmic proteomics showing differential expression of proteins after PHY34 (100 nM) treatment in OVCAR3 cells. **C** List of molecules and pathways from nuclear-cytoplasmic proteomics. **D** Representative immunoblots for validation of top hits from nuclear-cytoplasmic proteomics. **E** Gene set enrichment plots showing changes in p53 signaling pathway altered by PHY34 treatment in OVCAR3 and OVCAR8 cells. **F** Representative immunoblot for KPNA2 expression in HGSOC cells treated with PHY34 at various timepoints. **G** Representative immunoblot for p53 expression in nuclear and cytoplasmic protein fractions of cells treated with PHY34.

PHY34 did not change the expression and localization of CAS but did appear to shift the localization of an NLS-mCherry tagged reporter construct (Fig. 5C, D). The most well-studied role of CAS is nuclear-cytoplasmic transport specifically in exporting importins from nucleus [47]. To identify cargo mislocalized by PHY34 treatment, we prepared nuclear fractions in OVCAR3 cells treated with PHY34 and performed quantitative proteomics. Figure 6A–C shows the list of pathways and proteins impacted by PHY34 treatment. Many of these proteins were related to proliferation and DNA damage repair, such as PCNA, RAD51, and p53, as well as autophagy (LAMP1/2 and ACSS2). We validated histone H3, LAMP1/2, ACSS2, and PCNA by performing immunoblotting and found nuclear accumulation of these proteins after PHY34 treatment (Fig. 6D). However, none of these cargo proteins provided a reasonable mechanism for autophagy inhibition.

Since our proteomics revealed that PHY34 alters nuclear cargo, we performed RNA sequencing to further investigate transcriptome changes in cells treated with PHY34. Volcano plot depicting top up- and downregulated genes after PHY34 treatment is shown in Supplemental Fig. 4A, B. GSEA analysis of the differentially regulated genes demonstrated enrichment of genes involved in apoptosis and autophagy pathway (Supplemental Fig. 4C), consistent with our previous findings that PHY34 regulates autophagy and apoptosis [17]. We validated the RNA-Seq analysis by performing qPCR analysis for some of the top hits such as LAMP3, RelB, and IL6 (Supplemental Fig. 4D).

Interestingly, PHY34 treatment caused a significant change in the p53 pathway as shown by RNA-Seq analysis (Fig. 6E). RNA-Seq and immunoblotting showed reduced expression of KPNA2 (Karyopherin subunit alpha 2) after PHY34 treatment (Fig. 6F). KPNA2 interacts with CAS during nuclear-cytoplasmic transport, and KPNA2 loss is associated with autophagy inhibition due to cytoplasmic accumulation of wild-type p53 [47, 48]. In order to determine if PHY34 treatment changes p53 localization due to loss of KPNA2, we performed immunoblotting after nuclear-cytoplasmic fractionation. As shown in Fig. 6G, PHY34 treatment causes nuclear accumulation of mutant p53 in HGSOC cell lines. However, the established role for autophagy inhibition was cytoplasmic accumulation of wild-type p53 [47, 48], not the nuclear accumulation of mutant p53. CAS knockdown increased KPNA2 expression (data not shown), suggesting that PHY34 action on mutant p53 may not be related to CAS. Importantly, accumulation of mutant p53 may induce apoptosis due to activation of DNA damage pathway which was validated by γH2.AX expression in PHY34 treated cells (Supplemental Fig. 4E). This suggests that PHY34’s impact on nuclear cargo likely contributes to apoptosis instead of autophagy inhibition. Lastly, to compare the binding affinity between PHY34 and CAS, we expressed recombinant CAS protein and performed surface plasmon resonance (SPR) analysis. SPR data showed dose-dependent binding of CAS with PHY34 (Supplemental Fig. 4F), however, the amount of PHY34 required to saturate CAS was in high µM concentration. This was distinct relative to the nM concentrations of PHY34 required to induce apoptosis and inhibit autophagy indicating an alternate target of PHY34 in HGSOC cells.

### PHY34 inhibits V-ATPase activity via inhibition of the ATP6V0A2 subunit

**Fig. 7:**
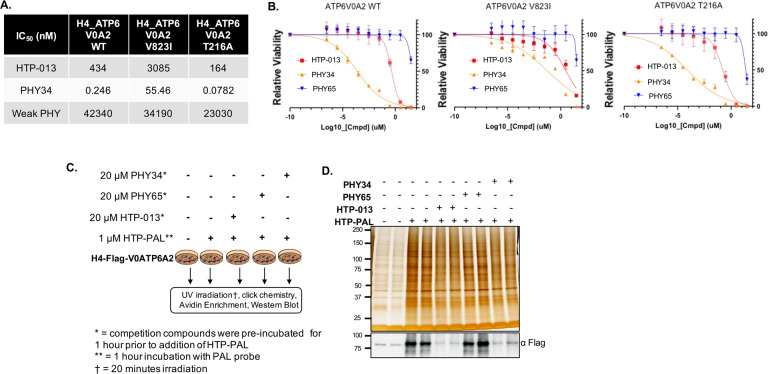
PHY34 inhibits V-ATPase activity via inhibition of the ATP6V0A2 subunit. **A** IC_50_ values (nM) for PHY34 mediated cytotoxicity in H4 cells expressing WT or mutant forms of the ATP6V0A2 subunit. **B** IC_50_ graphs demonstrating cell viability were obtained by performing CellTiter-Glo assay. **C** Schematic overview of photoaffinity labeling strategy using PHY34, PHY65, HTP-013, and HTP-PAL, a derivative of HTP-013. **D** (Top) Silver staining shows results of photoaffinity labeling pulldown. (Bottom) Immunoblot using Anti-Flag antibody to demonstrate binding between compounds and H4-Flag-V0ATP6A2.

Diphyllin compounds structurally similar to PHY34 have been proposed to function as V-ATPase inhibitors that block lysosomal acidification and autophagy [9–12]. A recent study showed a direct interaction between a diphyllin compound called HTP-013 and the ATP6V0A2 subunit using live-cell photoaffinity labeling-based chemoproteomics. PHY34 differs from HTP-013 from the combination of two key structural motifs: a C5''-hydroxymethyl and a C3'', C4''-acetonide. Modification in the sugar moiety in PHY34 enhances its potency, distinguishing PHY34 from previous analogs [16]. HTP-013 did not act as a topoisomerase 2 poison, bafilomycin A1 resistant cell lines were still sensitive to it (suggesting a unique mechanism), and mutants generated by error-prone PCR could produce specific amino acid alterations in the ATP6V0A2 subunit that conferred resistance [12]. In order to investigate if PHY34 has a similar mode of action as HTP-013, we used the previously reported wild type and mutant H4 cell lines [12]. As shown in Fig. 7A, B, PHY34 was 1000-fold more potent (246 pM vs 434 nM) than HTP-013, and mutation in V823I conferred resistance to both HTP-013 and PHY34, while mutation in T216A did not impact activity of either compound. These data indicate that PHY34 mimics HTP-013 in its ability to induce cell death in the presence of wild-type V0A2, but not V823I mutants. Photoaffinity labeling experiment revealed that both PHY34 and HTP-013 bind to the same site of ATP6V0A2 subunit. Interestingly, PHY65 did not show binding affinity to ATP6V0A2 subunit indicating binding specificity of PHY34 using an HTP-013-based photoaffinity probe (HTP-PAL) in a photoaffinity labeling experiment (Fig. 7C, D). HTP-PAL contains a diazirine cross-linker and a bioorthogonal alkyne moiety which allows covalent cross-linking between interacting proteins and compound upon UV irradiation [12, 49]. RNA-Seq analysis for PHY34 treated OVCAR3 and OVCAR8 cells showed reduced expression of ATP6V0A2 subunit in OVCAR3 cells and no change in expression for OVCAR8 cells, which was validated by qPCR analysis (Supplemental Fig. 5A, B). No significant change in ATP6V0A2 protein expression was observed in both OVCAR3 and OVCAR8 cells (Supplemental Fig. 5C). Additionally, RNA-Seq analysis found ABCA1 as one of the most highly downregulated transcripts (log fold 2.95 (OVCAR3) and log fold 2.1 (OVCAR8)). ABCA1 is a target known to be directly affected by V-ATPase inhibition [50].

## Discussion

This work reports on a compound that inhibits V-ATPase action and interacts with CAS to impact nuclear-cytoplasmic transport. Cancer cell lines and clinical samples provide support for CAS silencing to induce apoptosis in numerous cancers, including ovarian cancer [26–33, 35–37, 39, 51]. PHY34 interacted with CAS in two HGSOC cell lines, OVCAR8 and OVCAR3; however, interaction with ATP6V0A2 may have gone undetected for multiple reasons including compound orientation on the bead, lack of interaction with targets in lysosomal membrane, or ATP6V0A2 binding competence in cell lysate. CAS overexpression was able to reduce PHY34 induced apoptosis and cytotoxicity. PHY34 treatment caused a shift in the localization of NLS-tagged mCherry and a shift in the accumulation of specific nuclear proteins. However, the alteration of nuclear cargo could not fully explain autophagy inhibition, which was revealed to be through ATP6V0A2 inhibition, based on resistant mutants developed to test HTP-013 and confirmed by chemoproteomics. These data support that PHY34 is both an autophagy inhibitor and also a small molecule that binds to CAS shifting nuclear cargo proteins.

PHY34 bound CAS, in addition to other nucleocytoplasmic transport proteins, including importins: KPNB1 and KPNB2. Importazole is a KPNB1 inhibitor, and KPT-330 (known as selinexor) is an inhibitor of XPO1, the main exportin of the cell [52]. KPT-330 is in Phase 1, 2, or 3 clinical trials for a variety of cancers, including ovarian cancer, and achieved FDA Orphan Drug/Fast Track status for refractory multiple myeloma [52]. PHY34 activity appears to be distinct from these inhibitors in terms of in vitro potency and regulation of autophagy. Furthermore, XPO1 and CAS have different cargoes, and this formulates a rationale for personalized therapy in different cancers.

Other diphyllin structures have been reported to block lysosome acidification [9, 12, 53]. PHY34 is an ATP6V0A2 inhibitor with significantly higher inhibition potency than the previously reported compound HTP-013. Mutation of two residues in ATP6V0A2 conferred resistance to HTP-013, but not bafilomycin A1 or other autophagy inhibitors proving a novel mode of action [12]. Interestingly, at high concentrations, both sensitive and resistant mutants of ATP6V0A2 responded to PHY34, but not HTP-013 suggesting it might have another target, which we predict is CAS. This polypharmacology hypothesis would be in line with our data showing that CAS knockdown cells still undergo apoptosis in response to PHY34. SPR data with CAS suggested that the binding affinity of PHY34 was lower (µM) than what was predicted based on cell toxicity data (pM), suggesting an alternative target. These data support that PHY34 causes cell death due to autophagy inhibition by changing lysosomal acidification due to V-ATPase inhibition, but they provide some interesting questions to be tested regarding nuclear proteins that are the cargo of CAS. We found that PHY34 treatment reduced the RNA and protein expression of KPNA2. KPNA2 loss is associated with autophagy inhibition due to cytoplasmic accumulation of wild-type p53 [47, 48]. KPNA2 regulates subcellular localization of DNA damage response proteins p53, BRCA1, and Rad51 [54]. Hence, it is possible that PHY34 causes DNA damage in HGSOC cells due to aberrant localization of cargo proteins involved in DNA repair. Future studies will focus on defining the binding site of PHY34 with ATP6V0A2 and affinity studies in ovarian cancer cells.

Finally, we showed the importance of CAS in ovarian cancer clinical samples. Consistent with other cancers, we found that CAS is highly expressed in ovarian cancer, and its expression correlated with disease severity. As ovarian CNA and 20q13 amplification studies suggest [25, 55], we found CAS CNA gains in >60% of samples, which correlated with increased gene and protein expression. In conclusion, evidence acquired from clinical sample analysis reveals CAS to be a highly promising drug target. PHY34 is a small molecule inhibitor capable of binding CAS and its inhibition alters nuclear import and cell cycle, which was specific for cancerous over non-tumorigenic cells.

The ATP6V0A2 subunit is overexpressed in ovarian cancer, associated with cell membrane driving metastasis, and blocking its action was shown to overcome cisplatin resistance in ovarian cancer [6–8]. While we previously reported the ability of PHY34 to inhibit lysosomal acidification and reduce ovarian tumors, this work demonstrates PHY34 inhibits the ATP6V0A2 subunit [17]. While V-ATPase and autophagy inhibitors are desirable for cancer therapy, lead compounds with subunit specificity that could target tumors have been challenging to develop. Bisbenzimidazole derivatives were screened, but these compounds are active in the micromolar range [56]. BRD1240 is a diversity oriented synthetic V-ATPase inhibitor, but its activity profile overlapped significantly with bafilomycin A1 in the treated cell panels and it also demonstrated micromolar toxicity [57]. Hydroxychloroquine remains the primary late-stage autophagy inhibitor used in clinical trials and its use provides a route to enhance response to platinum therapy and immune-based therapy, making the identification of new bioavailable, highly potent V-ATPase inhibitors significant. PHY34 represents a highly potent cytotoxic small molecule against HGSOC, which functions to inhibit V-ATPase and CAS activity leading to autophagy inhibition.

## Materials and methods

### Compounds

Purchased compounds included chemotherapeutics [paclitaxel (Sigma #T7402)], early stage autophagy inhibitor [PI-103 (LC Labs #P-9099)], late-stage autophagy inhibitors [bafilomycin A1 (Sigma #SML1661, LC Labs #B-1080 in LC3B puncta and autophagic flux assays, and Cayman Chemical #11038 for cytotoxicity assays)] and chloroquine (Sigma #C6628), and nucleocytoplasmic transport pathway inhibitors [importazole (Cayman Chemical #21491) and KPT-330 (Cayman Chemical #18127)] [52, 58]. Structures of non-FDA-approved small molecules may be found here: bafilomycin A1 [59], importazole [60], and HTP-013 [12]. Photoaffinity labeled PHY analogs were created as previously published [17, 61]. All compounds were suspended in dimethyl sulfoxide (DMSO), and the final vehicle concentration < 0.1% (v/v).

### Cell culture

Ovarian cancer cell lines (OVCAR3, OVCAR4, OVCAR8), neuroglioma cells (H4), and transformed human embryonic kidney cells (HEK293T) were purchased from the American Type Culture Collection. Immortalized human ovarian surface epithelial cells (IOSE80) were a gift from Nelly Auersperg at the University of British Columbia (Vancouver, Canada), immortalized fallopian tube epithelial cells (FT33) were a gift from Ronny Drapkin at the University of Pennsylvania. eGFP-LC3B HeLa and mCherry-eGFP-LC3B HeLa cell lines were a gift from Ramnik Xavier at Massachusetts General Hospital. OVCAR3 and OVCAR4 were grown in RPMI1640 supplemented with L-glutamine (2 mmol/L), 10% fetal bovine serum (FBS, 100 I.U./mL), and 1% penicillin/streptomycin (P/S, 100 mg/mL). OVCAR8 cells, OVCAR8-RFP, H4, and HEK293T were grown in DMEM with 10% FBS and 1% P/S. IOSE80 was maintained in v/v 50% Medium 199 and v/v 50% MCBD with 15% FBS, 1% P/S, 1% l-glutamine, and 11 ng/mL epithelial growth factor. FT33 cells were grown in Advanced DMEM/F12 with 2% Ultroser G (Pall 15950-017) and 1% P/S. HeLa cells were cultured in DMEM with 8.8% FBS, 1.8% l-glutamine, and 1% P/S. All cultured cells were mycoplasma free and validated by short tandem repeat analysis in 2021. Cells were passaged a maximum of 20 times and maintained in a humidified incubator at 37 °C in a 5% CO_2_ environment.

### Plasmids and stable cell lines generation

CAS/CSE1L shRNA was cloned in pLKO.1 plasmid (Addgene plasmid #10878). pLenti-GIII-CMV-CSE1L-HA plasmid was purchased from Applied Biological Materials Inc. (Catalog # LV127800). pICE-NLS-mCherry, was a gift from Patrick Calsou (Addgene plasmid # 60364) [62]. Lentiviral particles were generated by transfecting HEK293T cells with third-generation lentiviral packaging constructs pCMV-VSV-G (Addgene plasmid #8454) and pCMVR8.74 (Addgene plasmid #22036). Viral supernatant was used to transduce cells using polybrene followed by puromycin (1 µg/ml) selection to generate single-cell clones. ATP6V0A2 wild-type and mutant cell lines were generated as previously described [12].

### Annexin V/propidium iodide staining

Cells were seeded in a 60-mm plate and allowed to attach overnight. Cells were treated with PHY34 and vehicle control (DMSO) for 24 h. Media was collected and cells were trypsinized and subjected to Annexin V-FITC/Propidium Iodide Apoptosis Assay (Nexcelom Biosciences) according to the manufacturer’s instructions. K2 Cellometer was used to detect fluorescence using FCS express software. Gating channels were applied as per the manufacturer’s protocol.

### Immunofluorescence analysis

Cells grown on glass coverslips were fixed using 4% paraformaldehyde, permeabilized with 0.2% Triton X-100, and blocked with 1% bovine serum albumin (BSA) in phosphate-buffered saline (PBS). Cells were then incubated with primary antibody (diluted in blocking solution) for 1 h at room temperature. The cells were washed with washing buffer (PBS with 0.05% Triton X-100) and then incubated with fluorescent secondary antibody (diluted in blocking solution) for 1 h at room temperature. Cells were washed three times with washing buffer. Actin was stained by phalloidin staining. Nuclei were stained with DAPI (0.1 mg/mL; Thermo Fisher Scientific # EN62248) for 10 min at room temperature. Cells were washed using washing buffer and coverslips were mounted on glass slides using mounting media (Vector Laboratories #H-1000). Images were acquired using 40X objective on a Nikon Eclipse E600 microscope using DS-Ri1 digital camera and NIS Elements Software (Nikon Instruments).

### Pulldowns assays

Initial pulldowns were conducted as outlined as published [61]. Lysates were collected in lysis buffer [50 mM Tris-HCl, 150 mM NaCl, 2 mM MgCl_2_, 2 mM CaCl_2_, pH 7.5, with protease inhibitor mixture (Roche)] after passage through a 27.5 gauge needle, freezing at −80 °C, and remove cellular debris with high-speed centrifugation for 15 min. Beaded PHY34 or negative controls were incubated overnight with cell lysates at 4 °C in polyethylene filtered, 10 μm spin columns (Pierce). Unbound proteins were removed with centrifugation (flow-through), and beads were washed three times with lysis buffer containing 0.3% Triton X-100 prior to elution in buffer [125 mM Tris-HCl, 10% 2-mercaptoethanol, 4% sodium dodecyl sulfate (SDS), 20% glycerol, 0.004% bromophenol blue, pH 6.8] followed by heating at 100 °C for 5 min. Eluates were separated by SDS-PAGE, proteins stained using Coomassie blue stain and gel bands excised for mass spectrometry analysis. In competition pulldown assays, unbeaded PHY34 was added to elution buffer for incubation in increasing concentrations (1 nM, 100 nM, and 10 μM) for 10 min each at 4 °C prior to heat elution.

### Mass spectrometry analysis of gel bands

Protein identification was carried out for each of the excised gel bands. Briefly, proteins in each gel band were reduced with 5 mM dithiothreitol at 55 °C for 20 min, alkylated with 15 mM iodoacetamide at room temperature for 20 min in the dark and enzymatically digested via trypsin at 37 °C overnight. Peptides from each gel band were extracted with 1:1 acetonitrile:0.1% formic acid (v/v), dried *in vacuo* and resuspended in 0.1% (v/v) formic acid. LC-MS analysis was done by using an Agilent 1290 Infinity II system coupled directly to an Agilent 6550 Q-TOF, where peptides were resolved using a 10 min increasing organic linear-gradient (mobile phases (A) 0.1% formic acid and (B) 100% acetonitrile with 0.1% formic acid). Raw mass spectrometry data was converted to a MGF using the Agilent Mass Hunter Qualitative Analysis (B.07.00) software. Proteins were identified using the Mascot (v2.6.2) after searching tandem spectra against the Swiss Protein *Homosapien* Database and results visualized in Scaffold (v4.8.7).

### Proteomic analysis of nuclear fractions

Nuclear fractions were isolated using NE-PER™ Nuclear and Cytoplasmic Extraction Reagents (Thermo Scientific™ #78833) from both the control and PHY34 treated OVCAR3 cells. Protein concentration was determined from nuclear fractions by Bradford assay (Bio-Rad #5000006). Next, 50 μg of protein from each nuclear extract was subjected to enzymatic digestion using the Mini S-Trap (ProtiFi) cartridge per the manufacturer’s protocol after being reduced with 10 mM dithiothreitol for 20 min at 55 °C and alkylated with 30 mM iodoacetamide for 20 min at room temperature. Peptides from each sample were isotopically labeled using an iTRAQ 8-plex kit and fractionated as previously described [63]. Peptide separation and mass detection occurred using a Q-Exactive mass spectrometer as previously described [64]. Raw data for the LC-MS analysis were searched against the Swiss Protein Homosapien database using the Proteome Discoverer (v2.3, Thermo Fisher, Carlsbad, CA) software. Here, trypsin was set as the protease with two missed cleavages, and searches were performed with precursor and fragment mass error tolerances set to 10 ppm and 0.02 Da, respectively. Peptide variable modifications allowed during the search were oxidation (M) and iTRAQ 8-plex (S, T, Y), whereas carbamidomethyl (C) and iTRAQ 8-plex (peptide N-terminus and (K)) and was set as fixed modifications. Differentially expressed proteins for PHY34 relative to control were determined by applying an unpaired *t*-test (*p* < 0.05). Biological relevance was determined for altered proteins using the Ingenuity Pathway Analysis software (IPA, Ingenuity Systems, Redwood City, CA) where a Right-tailed Fisher’s exact test was used to calculate a *p*-value to determine the probability for both pathways and disease and biological function analysis from the IPA Knowledge Base Library to those most significantly enriched.

### CAS protein expression and purification

pET21b vector expressing human CAS protein (NP_001307; 971aa) with a C-terminal His6-tag (Genscript) and transformed into BL21(DE3) cells. Protein expression was induced using an auto-induction protocol modified from Studier et al 2005 [65]. Cells were grown for 24 h at 25 °C and then harvested by centrifugation. All purification steps were performed at 4 °C, and protein yield at each step was monitored by Bradford assay. Frozen cell pellets were lysed by sonication in buffer A (20 mM Tris-HCl, pH 8.0, 0.5 M NaCl) containing 10 ug/mL lysozyme. The lysate was clarified by centrifugation and loaded onto a 2 mL HiTrap Talon crude column equilibrated with Buffer A. Bound CAS-His6 was eluted with a linear gradient of 0-150 mM imidazole in buffer A, and fractions containing CAS-His6 were pooled, concentrated, and exchanged into buffer B (20 mM HEPES, pH 7.5, 150 mM NaCl, 5 mM dithiothreitol). The protein was further purified by gel filtration chromatography on a Superdex 200 10/300 column equilibrated in Buffer B. Fractions containing CAS protein were pooled, concentrated to 1.5 mg/mL, and supplemented with 20% v/v glycerol before being flash frozen and stored at −80 °C.

### Surface plasmon resonance (SPR)

Purified CAS protein was diluted to 50 μg/mL with 10 mM sodium acetate (pH 4.25) and immobilized to a CM5 sensor chip by standard amine-coupling in PBSP buffer (10 mM phosphate, pH 7.4, 2.7 mM KCl, 137 mM NaCl, 0.05 % Tween-20) using a Biacore T200 instrument (GE Healthcare). Prior to protein loading, the CM5 sensor chip surface had been activated by a 1-ethyl-3-(3-dimethylaminopropyl) carbodiimide hydrochloride (EDC)/N-hydroxy succinimide (NHS) mixture. CAS protein was immobilized to flow channels 2 and 4 to ~10,000 RU, followed by ethanolamine blocking of the unoccupied surface area. Blank immobilization using EDC/NHS and ethanolamine was performed for flow channels 1 and 3, both reference channels. Compound solutions with a series of increasing concentrations (0.4, 0.8, 1.6, 3.1, 6.3, 12.5, 24, and 50 µM) were applied to the active and reference channels in SPR binding buffer (20 mM HEPES, pH 7.5, 150 mM NaCl, and 2% DMSO) at a 30 µL/min flow rate at 25 °C, with association and dissociation times of 60 and 120 s, respectively. Each dose response was performed in triplicate on all channels. The data were double referenced with reference channel and zero concentration (2% DMSO) responses, and reference-subtracted sensorgrams were fitted with various models using the Biacore evaluation software.

### Immunoblot analysis

Whole-cell lysates: Cells were lysed in RIPA lysis buffer [50 mM Tris pH 7.6, 150 mM NaCl, 1% Triton X-100, 0.1% SDS with protease (Roche) and phosphatase (Sigma) inhibitors], incubated at −80 °C, and centrifuged. For cell fractionation, nuclear and cytoplasmic protein fractions were isolated using NE-PER™ Nuclear and Cytoplasmic Extraction Reagents (Thermo Scientific™ #78833). Immunoblot analysis was performed as previously described [66]. Briefly, protein concentration was obtained using Bradford assay (Bio-Rad #5000205), and 20–30 μg protein per sample was separated by SDS-PAGE and transferred to nitrocellulose or activated 0.45 μm PVDF membranes (Thermo Fisher). After 5% milk block, the membranes were incubated with primary antibody (Table 1) overnight at 4 °C. Membranes were incubated with secondary antibody (Table 2) prior to visualization of signal with SuperSignal^TM^ West Femto substrate (Thermo Fisher) and imaging on a FluorChem E system (ProteinSimple).

**Table 1:** List of primary antibodies.

Antibody	Source	Dilution for WB	Dilution for immunofluorescence/immunohistochemistry
Anti-rabbit PARP	CST #9542	1:1000	–
Anti-rabbit LC3B	CST #2775	1:1000	–
Anti-rabbit GAPDH	CST #2118	1:10000	–
Anti-rabbit Actin	Sigma #A2066	1:5000	–
Anti-mouse CAS	sc-271537	1:1000	1:50
Anti-rabbit p53	CST #9282	1:1000	–
Anti-mouse KPNA1	sc-101292	1:1000	–
Anti-mouse KPNA2	sc-55538	1:1000	–
Anti-mouse KPNB1	sc-137016	1:1000	–
Anti-mouse KPNB2	sc-166127	1:1000	–
Anti-mouse LAMP1	CST #9091 T	1:1000	–
Anti-rabbit LAMP2	Proteintech 66301-1-Ig	1:1000	–
Anti-rabbit PCNA	CST #13110 S	1:1000	–
Anti-rabbit HDAC2	sc-81599	1:5000	–
Anti-mouse ACSS2	sc-398559	1:1000	–
Anti-rabbit H3	CST #9715	1:5000	–
Anti-γH2A.X	CST #9718 S	1:1000	–
Anti-DYKDDDDK Tag	CST #2368	1:1000	–
Anti-ATP6VOA2	ab96803	1:1000	–
Alexa FluorTM 594 Phalloidin	Invitrogen A12381	–	1:100

**Table 2:** List of secondary antibodies.

Antibody	Source	Dilution for WB	Dilution for immunofluorescence
Anti-rabbit IgG-HRP	CST #7074	1:10000	–
Anti-mouse IgG-HRP	CST #7076	1:10000	–
Anti-rabbit Alexa Fluor 488	Invitrogen# A-11034	–	1:1000

### RNA-sequencing

RNA was isolated from PHY34 and vehicle (DMSO)-treated cells using Qiagen RNAeasy Mini kit (#74104) as per the manufacturer’s protocol. RNA libraries (three technical replicates/treatment) were created. RNA quality determination, mRNA enrichment, library construction, sequencing, alignment, and gene expression summarization were performed at the Genomics Core Facility at Northwestern University. The summarized counts' data was compared between samples treated with PHY34 and DMSO control using R package limma with Voom transformation [67]. A controlling the number of mean false positives method [68] was used to adjust for multiple comparisons to control Type I error at 0.05.

### cDNA synthesis and qRT-PCR analysis

Total RNA (1 µg) was converted to cDNA using iScript cDNA Synthesis Kit (Bio-Rad). qRT-PCR measurements were performed using the CFX connect Real-Time PCR Detection System (Bio-Rad) and SYBR Green (Roche) according to the manufacturer’s protocol. Samples were normalized to the housekeeping gene, GAPDH. qRT-PCR primer sequences are mentioned in Table 3.

**Table 3:** List of qRT-PCR primers.

Target gene	Forward primer sequence (5'-3')	Reverse primer sequence (5'-3')
*LAMP3*	TAAAAGCAGAGATGGGGATAC	ATTTTCGGGTGCCACAGTTC
*IL6*	CCACTCACCTCTTCAGAACG	CATCTTTGGAAGGTTCAGGTTG
*RELB*	TCCCAACCAGGATGTCTAGC	AGCCATGTCCCTTTTCCTCT
*ATP6V0A2*	GCAGGAGTATGTCCAGAGAGTGC	GGTTCACCCCGAAGCAACTACG
*GAPDH*	ATGGGGAAGGTGAAGGTCG	GGGGTCATTGATGGCAACAATA

### Cell viability assay

Cells were seeded in 96-well, clear, flat-bottomed plates at 2500 to 5000 cells per well, depending on the cell line, and allowed to attach overnight. Compounds suspended in DMSO were diluted to final concentrations and added to the cells. The final vehicle concentration was 0.25% to achieve a wide dose range. Cells were incubated for 24, 48, or 72 h. Cellular protein content was measured using a sulforhodamine B (SRB) assay as a measure of cell survival [69]. Treatment measurements were normalized to vehicle, and dose-response curves with corresponding IC_50_ values were generated using GraphPad Prism Software.

### CellTiter-Glo assay using ATP6V0A2 mutant H4 cells

H4 cells expressing wild type and mutant forms of the ATP6V0A2 subunit were used to perform cell viability assay as previously described [12]. Cells were plated at a density of 1500 cells/well in 384-well plates and treated with compounds for 3 days. Compounds were serially diluted 1:10 from 30 µM to 3 pM. Cells were treated in quadruplicate. As a positive control, cells were treated with 30 µM MG-132. Vehicle control was set to 100% cell viability and MG-132 treatment was set to 0% cell viability. Cell viability was quantified with the CellTiter-Glo assay (Promega), and luminescence was measured on an EnVision 2104 Multilabel reader. Treatment measurements were normalized to vehicle, and dose-response curves with corresponding IC_50_ values were generated using GraphPad Prism Software.

### Photoaffinity label pulldown

Photoaffinity labeling experiments with an HTP-013-based photoaffinity probe were performed as previously described [12]. Silver staining was performed using Pierce™ Silver Stain for Mass Spectrometry kit (Catalog#:24600) according to the manufacturer’s protocol.

### LC3B puncta and autophagic flux assay

HeLa cells stably expressing eGFP-LC3 (LC3 puncta assay) or stably expressing mCherry-eGFP-tagged LC3 (autophagic flux assay) [57] were seeded in 384-well plates at 3000 cells per well. After attachment overnight, test compounds and controls were moved into assay plates using a 96-well pin tool (V&P Scientific) and a liquid handler, Biomek NXP Lab Automation Workstation (Beckman Coulter). Assay plates were incubated at 37 °C for 4 (LC3 puncta assay) or 24 (autophagic flux assay) h. Cells were then fixed with w/v 4% formaldehyde (Thermo Scientific) in PBS for 12 min, washed with PBS, DNA-stained with 2 μg/mL Hoechst 33342 (Molecular Probes) in PBS for 12 min, and diluted with PBS. Plates were sealed using PlateMax Semi-Automatic Plate Sealer (Axygen). Plates were imaged at 10X by an automated fluorescence microscope, ImageXpress Micro XLS (Molecular Devices), using DAPI and FITC filters (both assays) and Texas Red filter for the autophagic flux assay. Four sites were imaged per well. The number of puncta per cell was quantified using the MetaXpress High-Content Image Analysis Software with Transfluor Application Module (Molecular Devices). The average number of autophagosomes (eGFP + /mCherry + ; yellow puncta) and autolysosomes (eGFP-/mCherry + ; red puncta) per cell was quantified using CellProfiler Software (Broad Institute). Treatment measurements were normalized to vehicle, and dose-response curves with corresponding EC_50_ values were generated using GraphPad Prism Software.

### Tissue microarray immunohistochemistry & scoring

Tissue sections were stained on BondRX autostainer (Leica Biosystems) following a preset protocol. In brief, sections were deparaffinized with Bond Dewax solution and subjected to EDTA-based (Bond ER2 solution, pH9) antigen retrieval for 40 min at 100 °C. Sections were blocked for 5 min with hydrogen peroxide block and washed with Bond Wash Solution. Following 30 min incubation with Background Sniper protein block (#BS966, Biocare Medical), sections were incubated with anti-CAS/CSE1L mouse monoclonal antibody (clone H-2, 1:50, Santa Cruz Biotechnology) antibody for 30 min. The detection was performed using Bond Polymer Refine Detection kit (Leica Biosystems DS9800) using the following conditions: post primary antibody incubation time and polymer-HRP incubation time were set to 15 min each, and DAB incubation time was 10 min. All slides were counterstained with hematoxylin for 5 min and mounted with Surgipath Micromount Media (Leica Biosystems). CAS antibody was optimized on human placenta samples and validated using CHTN Test TMA, which included various tissue types positive (placenta, colon, and breast) and negative (liver, spleen, and uterus) for CAS. Secondary antibody only control confirmed the specificity of the staining.

### Statistical analysis

Data presented are the mean ± standard error of the mean (SEM) and represent at least 3 independent experiments. Statistical analysis was carried out using GraphPad Prism software. For the in vitro cell line experiments, two-sample *t*-tests were used for two groups and ANOVA were used for multiple group comparisons respectively with Dunnett’s, Holm’s, or Tukey’s adjustment for multiple comparisons to control family-wise type I error at 0.05, as noted in legends. Log-rank test was used for survival analysis. Pearson Correlation was used to test the correlation between gene expression and CIN70 score. Pathway analysis was performed with Gene Set Enrichment Analysis (GSEA). Adjusted *p* < 0.05 is considered significant with stars denoting significance as follows: *p < 0.05, **p < 0.01, ***p < 0.001, and ****p < 0.0001.

## Supplementary information

Supplemental data file

Reproducibility checklist

## Data Availability

The authors confirm that the data supporting the findings of this study are available within the article and its Supplementary material. Raw data that support the findings of this study are available from the corresponding author, upon reasonable request.
