# Supplementary material for: PHY34 inhibits autophagy through V-ATPase V0A2 subunit inhibition and CAS/CSE1L nuclear cargo trafficking in high grade serous ovarian cancer
Source: Cell Death Dis. 2022 Jan 10;13(1):45. doi: 10.1038/s41419-021-04495-w (PMC8748433; doi:10.1038/s41419-021-04495-w)
Supplement: Supplementary file 1 — Supplemental data file [file 41419_2021_4495_MOESM1_ESM.pdf]

Supplemental data

Supplemental Figure 1

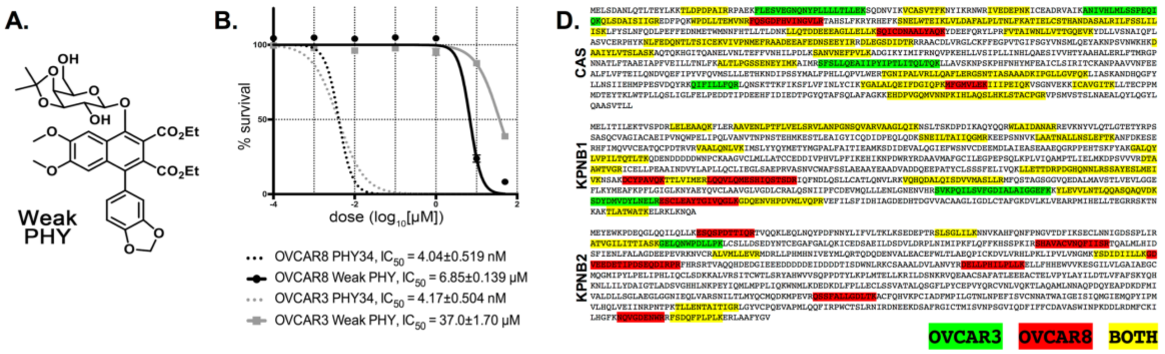

C.

	OVCAR8				OVCAR3			
	Number of Biological Reps. Found In	Unique Peptide Count	Percent Protein Coverage	Highest Probability of Identification	Number of Biological Reps. Found In	Unique Peptide Count	Percent Protein Coverage	Highest Probability of Identification
KPNB2/TNPO1	3/3	12	15.6%	100%	3/3	7	8.3%	100%
IPO4	2/3	14	15.0%	100%	3/3	21	26.2%	100%
IPO7	2/3	15	17.0%	100%	3/3	21	25.9%	100%
IPO5	2/3	22	24.2%	100%	2/3	12	13.5%	100%
IPO9	2/3	10	8.6%	100%	2/3	9	11.0%	100%
XPOT	1/3	4	4.1%	100%	2/3	5	5.5%	100%

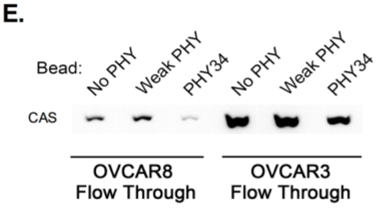

Supplemental Figure 1.

A. Structure of Weak PHY (PHY65).

B. Dose response curves and IC_{50}s displaying Weak PHY's micromolar activity compared to PHY34 in HGSOC cell lines, OVCAR8 and OVCAR3.

C. Other targets identified by mass spectrometry, which appeared in multiple biological replicates and in both HGOSC cell lines; all are members of the nucleocytoplasmic transport pathway.

D. Visualization of percent protein coverage per target with peptide hits color-coded by the HGSOC line they were identified by mass spectrometry in (OVCAR8=red, OVCAR3=green, both cell lines=yellow).

E. Representative immunoblot assessing CAS amount in flow through (lysates incubated with beads prior to washing steps) of competition pulldown assay in HGSOC cell lines.

Supplemental Figure 2

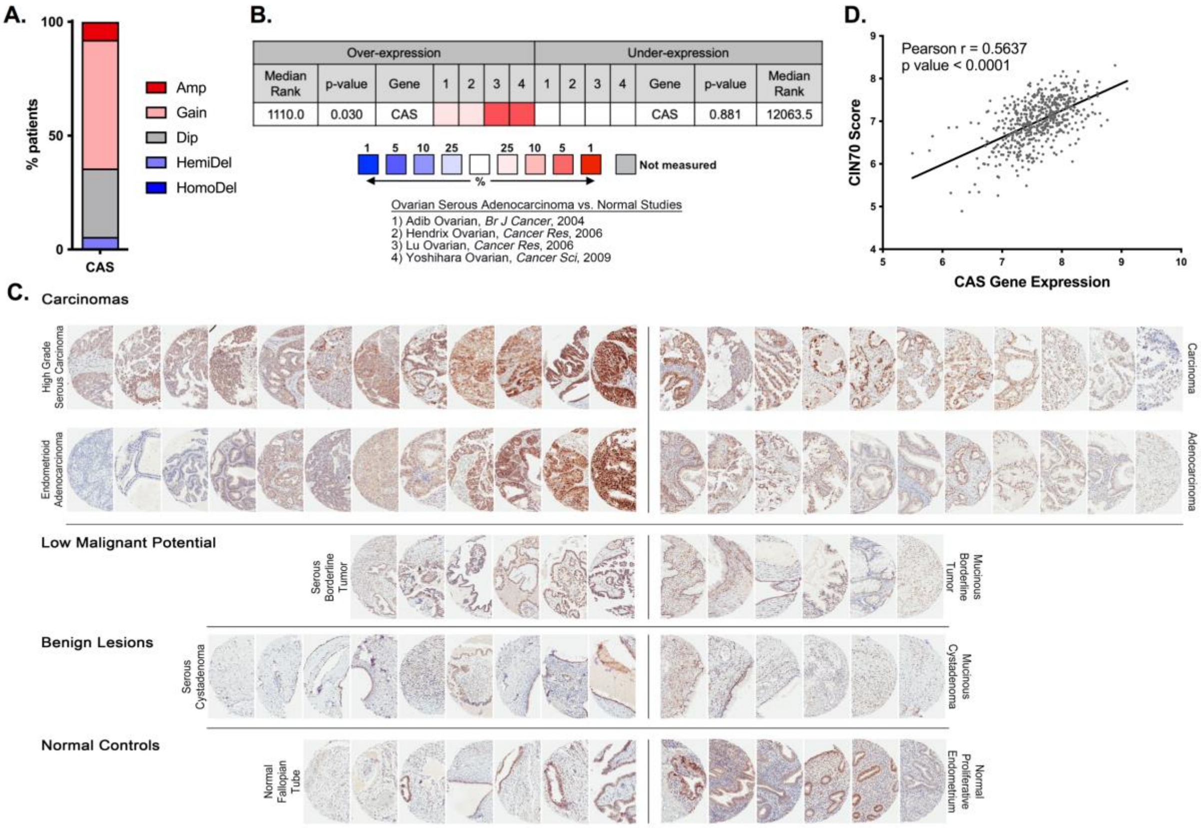

Supplemental Figure 2.

A. Copy number alterations (CNAs) in ovarian cancer patient tumors in the Cancer Genome Atlas (TCGA) database (n=579) for CAS.

B. Oncomine analysis of available ovarian cancer studies, comparing tumor expression data to normal controls (cited in figure).

C. CAS expression correlated to a measure of chromosomal instability, the CIN70 score, using TCGA ovarian cancer U133 microarray data (n=531). The linear relationship was assessed using the Pearson correlation coefficient and p value.

D. All TMA samples stained for CAS. Each half core represents one patient. TMA was obtained from the Cancer Human Tissue Network.

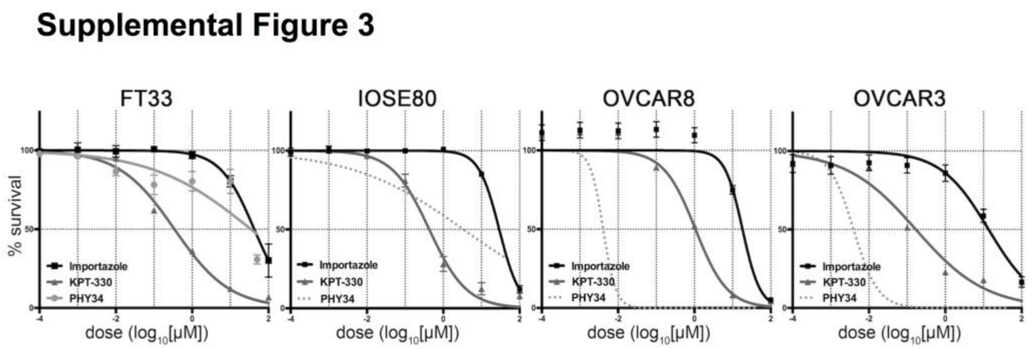

Supplemental Figure 3.

Dose response curves measuring cell viability after 72 hours treatment in HGSOC cell lines, OVCAR8 and OVCAR3, and non-tumorigenic cell lines, FT33 and IOSE80. Dotted lines display previously published data.

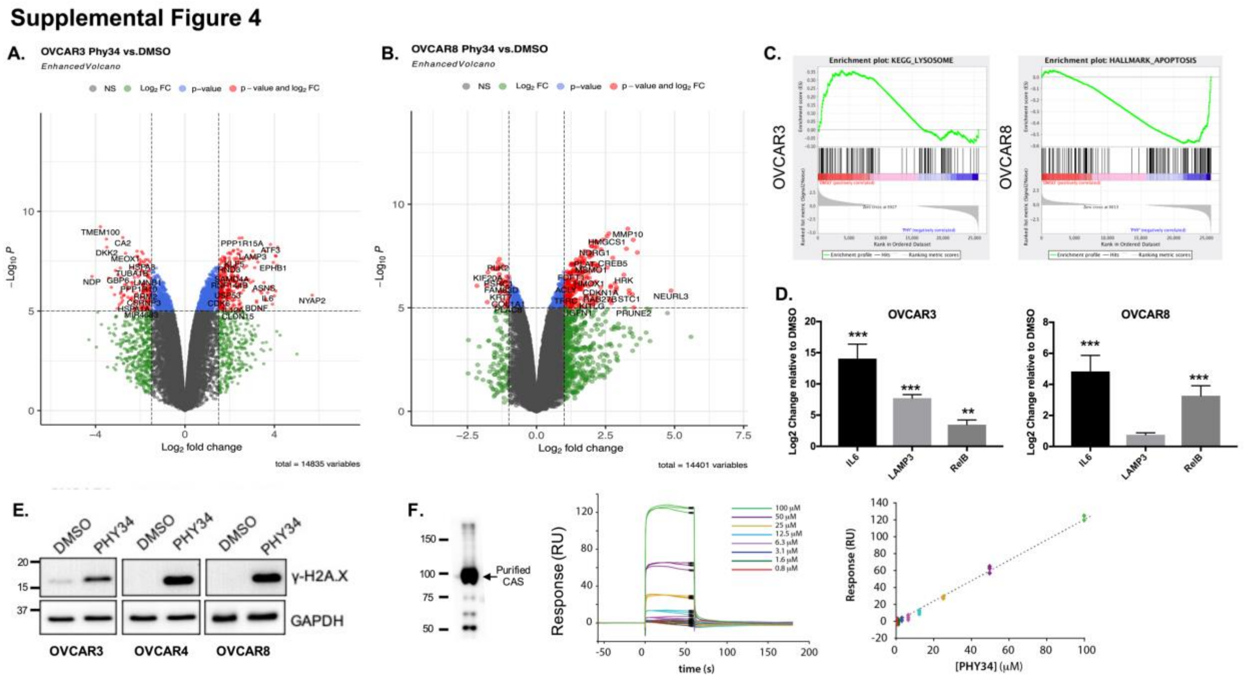

Supplemental Figure 4.

A. Volcano plot showing up-and downregulated genes by RNA-Seq analysis for vehicle and PHY34 (100 nM) treated OVCAR3 cells.
B. Volcano plot showing up-and downregulated genes by RNA-Seq analysis for vehicle and PHY34 (10 nM) treated OVCAR8 cells.
C. Gene set enrichment plots showing changes in apoptosis and autophagy pathway altered by PHY34 treatment in OVCAR3 and OVCAR8 cells.
D. Validation of differentially expressed genes IL6, RelB and LAMP3 by qPCR in OVCAR3 and OVCAR8 cells. Data represent mean ± standard error of the mean

(SEM). Statistics were generated with Student’s t-test. ** represents p<0.01, *** represents p<0.001.

E. Representative immunoblot for γ-H2A.X expression in OVCAR3, OVCAR4, and OVCAR8 cells treated with PHY34 (100 nM, 100 nM, and 10 nM, respectively).

F. Purified CAS protein was used to check binding with PHY34 in an SPR assay. SPR graph showing binding of CAS with various concentrations of PHY34 as a function of time.

Supplemental Figure 5

A. **OVCAR3**

Gene Symbol	Description	Log2 Fold Change	p Value	FDR Adj p Value	Significant	DMSO	PHY34
ATP6V0A2	ATPase H+ transporting V0 subunit a2	0.669292939	9.79E-34	8.81E-33	Yes	844.4282127	1344.039453
ATP6V0A1	ATPase H+ transporting V0 subunit a1	-0.319318329	0.00071908	0.001383	Yes	357.8559977	285.5260354
ATP6V0A4	ATPase H+ transporting V0 subunit a4	-0.15863415	0.72370478	NA	No	5.948778017	4.919825347

OVCAR8

Gene Symbol	Description	Log2 Fold Change	p Value	FDR Adj p Value	Significant	DMSO	PHY34
ATP6V0A2	ATPase H+ transporting V0 subunit a2	-0.00494316	0.9422302	0.964678	No	764.3880938	761.7148777
ATP6V0A1	ATPase H+ transporting V0 subunit a1	0.502510347	1.45E-22	2.64E-21	Yes	1102.464661	1567.001988
ATP6V0A4	ATPase H+ transporting V0 subunit a4	0.650985122	5.06E-15	5.82E-14	Yes	351.1137932	557.1665785

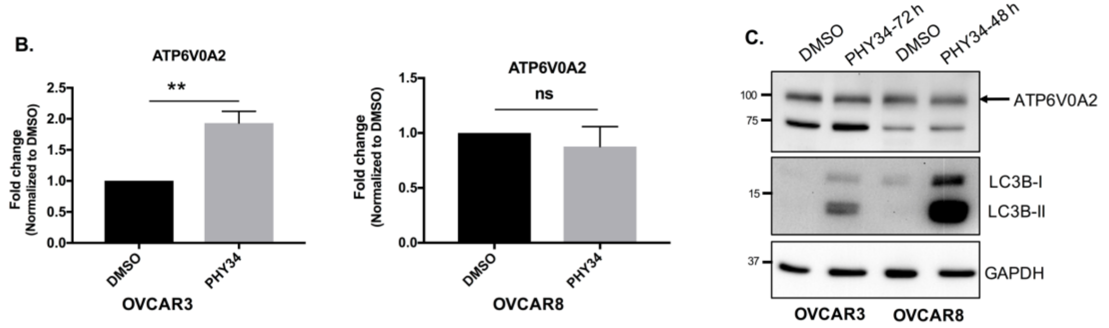

Supplemental Figure 5.

A. RNA-Seq analysis for ATP6V0A subunits in OVCAR3 and OVCAR8 cells.

B. Validation of ATP6V0A2 expression by qPCR in OVCAR3 and OVCAR8 cells treated with PHY34 (100 nM and 10 nM respectively). Data represent mean ± standard error of the mean (SEM). Statistics were generated with Student’s t-test. ** represents p<0.01, ns represents 'not significant'.

C. Representative immunoblot for ATP6V0A2 subunit expression in OVCAR3 and OVCAR8 cells treated with PHY34 (100 nM and 10 nM respectively).
